# STRUCTURAL DISCRIMINATION AND MRI-ASSESSED BRAIN ENDPOINTS: HANDLS BRAINCHILD

**DOI:** 10.1093/geroni/igz038.049

**Published:** 2019-11-08

**Authors:** Danielle L Beatty Moody, Rao P Gullapalli, Christos Davatzikos, Shuyan Sun, Lesile Katzel, Alan Zonderman, Michele Evans, Shari R Waldstein

**Affiliations:** 1 University of Maryland Baltimore County, Baltimore, Maryland, United States; 2 University of Maryland, School of Medicine, Baltimore, Maryland, United States; 3 University of Pennsylvania, Philadelphia, Pennsylvania, United States; 4 NIH/NIA, Intramural Research Program, Baltimore, Maryland, United States

## Abstract

Emerging evidence demonstrates that exposure to race-related adversity, specifically, individual-level discrimination, in middle-age is adversely linked with white matter lesion volume, a prospective marker of future cerebrovascular disease as indicated on Magnetic Resonance Imaging (MRI). It remains unclear whether exposure to indices of neighborhood-level structural discrimination (e.g., residential segregation, % of population employed & with high school diploma/equivalency), are linked to MRI-assessed brain pathology and how these linkages may be patterned by key sociodemographic characteristics (e.g., race, age, sex, class). Knowledge of this linkage may help us further understand well-documented racial disparities in multiple clinical brain health endpoints including stroke, dementia, cognitive decline, functional disability, and subclinical brain pathology in adulthood. Thusly, this talk will focus on work that examines whether neighborhood-level structural discrimination is associated with MRI-brain assessed indicators of subclinical brain pathology and the role of key sociodemographic factors, with emphasis on the role of race.

